# Effect of *Polygonum Multiflorum Thunb* on liver fatty acid content in aging mice induced by D-galactose

**DOI:** 10.1186/s12944-019-1055-y

**Published:** 2019-06-01

**Authors:** Jiangquan Yang, Yuqi He, Jiayi Zou, Lin Xu, Fang Fan, Zhenglong Ge

**Affiliations:** 1 Department of Biochemistry and Molecular Biology, Zunyi Medical University, Zunyi, Guizhou Province China; 2 Department of Pharmaceutical Analysis, Zunyi Medical University, Zunyi, Guizhou Province China

**Keywords:** *Polygonum Multiflorum Thunb*, Aging, Fatty acids, Gas chromatography mass spectrometry, D-galactose

## Abstract

**Background:**

*Polygonum Multiflorum Thunb*(PMT) has multiple biological effects, such as anti-inflammatory, lipid-lowering, anti-aging and so on. Therefore, D-galactose-induced aging mice were used to study the effect of PMT on fatty acid metabolism and its underlying mechanism.

**Methods:**

C57BL/6 male mice were randomly divided into normal group, aging model group, PMT intragastrical administration group (high, Medium, low); model group and PMT intragastrical administration group Daily intraperitoneal injection D-galactose 800 mg·ml^− 1^·Kg^− 1^ to establish subacute aging model; PMT intragastrical administration group at the same time to intragastrical PMT extract (1 g·ml^− 1^·Kg^− 1^, 0.6 g·ml^− 1^·Kg^− 1^, 0.3 g·ml^− 1^·Kg^− 1^), normal group injection and intragastrical equivalent saline for 60 consecutive days. By detecting the oxidation index of liver to judge the efficacy of PMT, gas chromatography-mass spectrometry (GC-MS) analysis was used to quantitatively analyze the fatty acid content in liver.

**Results:**

Finally, we found that PMT improved the enzyme activity of superoxide dismutase (SOD) and glutathione peroxidase (GSH-Px) in aging mice, and reduce the enzyme activity of malondialdehyde (MDA), aspartate aminotransferase (AST) and alanine aminotransferase (ALT). The content of fatty acids such as C18:1, C18:2, C18:3 N3, C20:2 and C20:3 N3 decreased significantly in senescent mice (*P* < 0.05) as evidenced by GC-MS analysis, whereas, these fatty acids increased significantly after treatment of PMT (*P* < 0.05).

**Conclusion:**

PMT improves the content of liver fatty acids in aging mice induced by D-galactose through, enhancing the activity of anti-oxidant enzymes.

## Introduction

Aging is often manifested in the degeneration of multiple tissues and organs of the body, abnormal function and other changes. Studies have shown that aging is often associated with cognitive dysfunction, oxidative stress injury, inflammation, etc. [1–3]. Lipids, as essential components of the body, often change in abnormal changes in the aging process [4, 5], and lipid metabolism plays a key role in the aging process [6, 7]. At present, the occurrence of aging cannot be inhibited, but it can be controled, delayed using drugs, such as *Polygonum Multiflorum Thunb* (PMT), which was widely used as a traditional chinese medicine for its anti-aging, anti-inflammatory antioxidant effect, et al. [8, 9]. PMT contains a variety of chemical constituents, of which tetrahydroxystilbene glucoside are one of its main components. Studies have shown that tetrahydroxystilbene glucoside can increase the expression of Klotho protein in nerve cells, reduce the way insulin, IGF-1 and IGF-1R, and prolong the life span of SAMP8 aging mice [10]. And tetrahydroxystilbene glucoside will increase the expression of SIRT1, the corresponding stress conditions of the tissue has a protective effect, can enhance the body tissue SOD and GSH-Px and other enzyme activity [11]. However, it remains unclear how PMT can delay aging and the effects of lipid changes during aging. Therefore, this study was designed to the effects of PMT on D-galactose aging mice and elucidate its possible molecular mechanism.

## Materials and methods

### Experimental animals

8-week-old SPF C57BL/6 male mice, purchased in Changsha Tian Qin Biotechnology Company Limited, license number: SCXK (Xiang) 2014–0011, weight 20 ± 2 g, free diet, lighting 12 h alternating lighting. Adaptive feeding for 4 days, randomly divided into normal group (C), aging model group (D), PMT intragastrical administration group (high, medium and low; PMT-H, PMT-M and PMT-L) (*n* = 6). The D group, PMT-H, PMT-M and PMT-L groups used D-galactose (800 mg·ml^− 1^·Kg^− 1^) peritoneal injection to establish the subacute senescence model, and the C group injected the same amount of saline. At the same time, PMT-H, PMT-M and PMT-L Groups daily use of PMT extract (1 g·ml^− 1^·Kg^− 1^, 0.6 g·ml^− 1^·Kg^− 1^, 0.3 g·ml^− 1^·Kg^− 1^) to intragastrical, the C group and the D group of intragastrical equivalent saline, continuous injection and intragastrical for 60 days. 4% of Chloral Hydrate peritoneal injection mice, each 100 g body weight injection 1 ml chloral hydrate. After the anesthesia of mice, broken head to kill mice, selected liver to detect corresponding indicators and fatty acid content analysis. Animal experiments were approved by the Animal Experiment Center of Zunyi Medical University, Zunyi, Guizhou province.

### Preparation of extraction fluid from PMT

PMT was purchased in the pharmacy of Zunyi Medical University affiliated hospital; Origin: Sichuan; Production Batch Number:161201. Take 100 g of PMT powder, pass the No. 4 sieve, add 5 times of 75% ethanol reflux 2 times, each time for 1 h, centrifuge to take the supernatant, and combine the two supernatants, the equivalent of raw 1 g·ml^− 1^ was concentrated by rotating evaporator. The content of 2,3,5,4′-tetrahydroxystibene-2-O-β-D-glucoside was detected by reference to Chinese Pharmacopoeia Method (fifth edition).

### Reagents and instruments

SOD, MDA, ALT, AST and GSH-Px assay kits (Nanjing jiancheng Bioengineering Institute, China); D-galactose (sigma, America); Hexane (Sinopharm Chemical Reagen Co. Ltd., China); Fatty acid internal standard methyl salicylate (TCI, China); Chloroform (Wokai, China); ddH2O (mini, Arium); sulfuric acid (Sinopharm Chemical Reagen Co. Ltd., China); Methanol (Sinopharm Chemical Reagen Co. Ltd., China); NU-CHEK-PREP 37 fatty acid methyl ester mixture (NU-CHEK-PREP, America); Agilent 6890 N/5975B Gas Phase-mass Spectrometry Combination Instrument (Agilent, United States); agilentHP-INNOWAX Capillary chromatography Column (Agilent, United States); Xiangyi Refrigerated Centrifuge (Xiangyi Instruments Co. Ltd., China); Eddy instrument (Haimen Kylin-Bell Lab Instruments Co. Ltd., China); Electric thermostatic water bath (Beijing Changan Science Instruments Co. Ltd., China); enzyme-labeled instrument (IMARK, America); Visible spectrophotometer (Inesa Analytical Instruments Co. Ltd., China).

### Determination of liver sample index

Take liver tissue on ice homogenization and centrifuged using 3500 RPM. Supernatant is collected gently and kept in − 20 °C until the activity of SOD, MDA, ALT, AST and GSH-Px is measured. The SOD was determined by xanthine oxidase method; ALT and AST were detected directly by enzyme marker; MDA was determined by the method of thiamine barbiturates (TBA); and the GSH-Px was determined by colorimetric method. The reagent solution was prepared according to the kit instructions, and the enzyme activity was detected and calculated by enzyme marker and type 722 ultraviolet spectrophotometer.

### GC-MS analysis of liver samples

#### Fatty acid standard configuration

NU-CHEK-PREP 37 Fatty acid methyl ester mixed solution (1000 μg·ml^− 1^) was used as the external standard. The standard curve is 1000 mg·L^− 1^, 500 mg·L^− 1^, 250 mg·L^− 1^, 100 mg·L^− 1^, 50 mg·L^− 1^, 25 mg·L^− 1^, 10 mg·L^− 1^, 5 mg·L^− 1^, 1 mg·L^− 1^ of nine concentration gradients, of which the concentration is the total concentration of each component. Of the 37 fatty acid methyl ester standard products, the concentration of each component as a proportion of the total concentration has a 2.63, 5.26% of two gradients.

#### Liver sample pretreatment (methyl ester)

The PMT-M Group, D group and C group with the best effect of the index were selected, and 3 of each group was analyzed by GC-MS. Take tissue 50 mg, add 1% sulfuric acid-methanol solution 2 mL, fully mix 1 min, put on 80 °C water bath, methyl ester half an hour, then add 1 mL N-hexane extraction, add 5 mL pure water washing, absorb supernatant 500 μl, add 100 mg anhydrous sodium sulfate to remove excess water, take supernatant to add internal standard 25 μl, Oscillation before entering the sample.

#### GC-MS analysis conditions

##### Chromatographic conditions

Chromatographic Column agilentHP-INNOWAX Capillary column (30 m*0.25 mm ID*0.25 μm); Shunt into the sample, the sample amount of 1 μl, not shunt. Inlet sample temperature 280 °C; Ion source temperature 230 °C; Transmission line temperature 25 °C. The program starts at a temperature of 50 °C, maintains 3 min, 10 °C·min^− 1^ to 220 °C, maintains 3 min, and finally rises to 250 °C with a 15 °C·min^− 1^, maintaining 10 min. Carrier gas is helium, carrier airflow speed 1.0 mL·min^− 1^.

##### MS conditions

Electronic Bombardment Ionization (EI) source, SIM scanning mode, electronic energy 70 eV.

### Data processing and analysis

All data were expressed as means ± standard deviation and analyzed using the SPSS 19.0. A *P*-value< 0.05 was considered to indicate a statistically significant result.

## Results

### Activity detection of SOD, MDA, ALT, AST and GSH-Px in liver tissue

Compared with the C group, the activity of SOD, GSH-Px in liver tissue of D group decreased obviously (*P* < 0.05), and the content of ALT, AST and MDA increased obviously (*P* < 0.05); Compared with the D group, the activity of SOD in liver tissue of PMT-H and PMT-M groups increased obviously (*P* < 0.05), significantly reduced the content of MDA, ALT and AST in the liver (*P* < 0.05); and significantly increased the activity of GSH-Px in the PMT-M and PMT-L groups (*P* < 0.05) (Table 1).

**Table 1:** Effects of PMT on SOD, MDA, ALT, AST and GSH-Px activity in the liver of senile mice (X̅ ± S, *n* = 6)

Groups	Dose(g·ml^− 1^·Kg^− 1^)	SOD(U·mg^− 1^)	MDA(U·mg^− 1^)	ALT(U·g^− 1^)	AST(U·g^− 1^)	GSH-PX(U·mg^− 1^)
C	–	50.57 ± 3.15	4.28 ± 1.48	65.88 ± 6.55	43.35 ± 5.28	141.61 ± 24.51
D	–	43.57 ± 5.96^#^	6.49 ± 2.08^#^	87.04 ± 6.4^#^	52.53 ± 4.03^#^	110.2 ± 15.64^#^
PMT-H	1	52.53 ± 2.38^*^	4.71 ± 0.91^*^	73.85 ± 8.49^*^	40.76 ± 2.3^*^	121.75 ± 26.61
PMT-M	0.6	51.98 ± 3.57^*^	4.21 ± 1.64^*^	70.7 ± 9.93^*^	43.28 ± 6.23^*^	163.65 ± 17.98^*^
PMT-L	0.3	49.35 ± 5.15	4.77 ± 0.53^*^	83.21 ± 4.49	49.1 ± 2.92	153.07 ± 16.55^*^

^#^ *P*<0.05 vs C group;* *P*<0.05 vs D group

### Full scan chromatogram of standard and liver samples

**Fig. 1:**
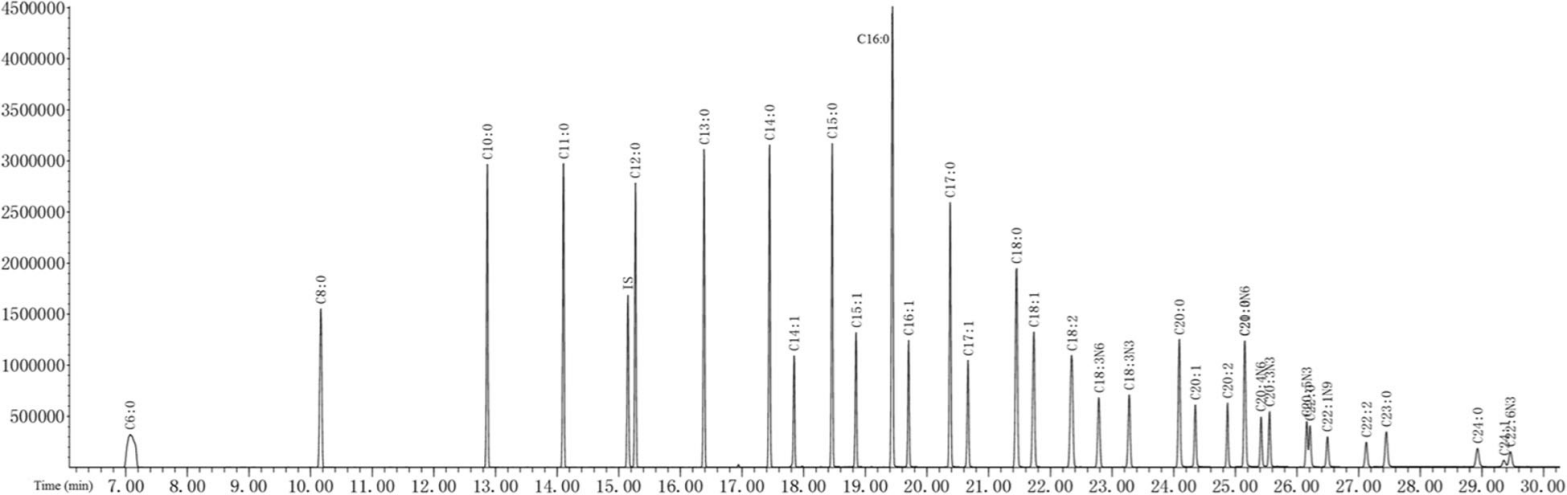
Total ion flow chromatography for fatty acid standard products (TIC)

**Fig. 2:**
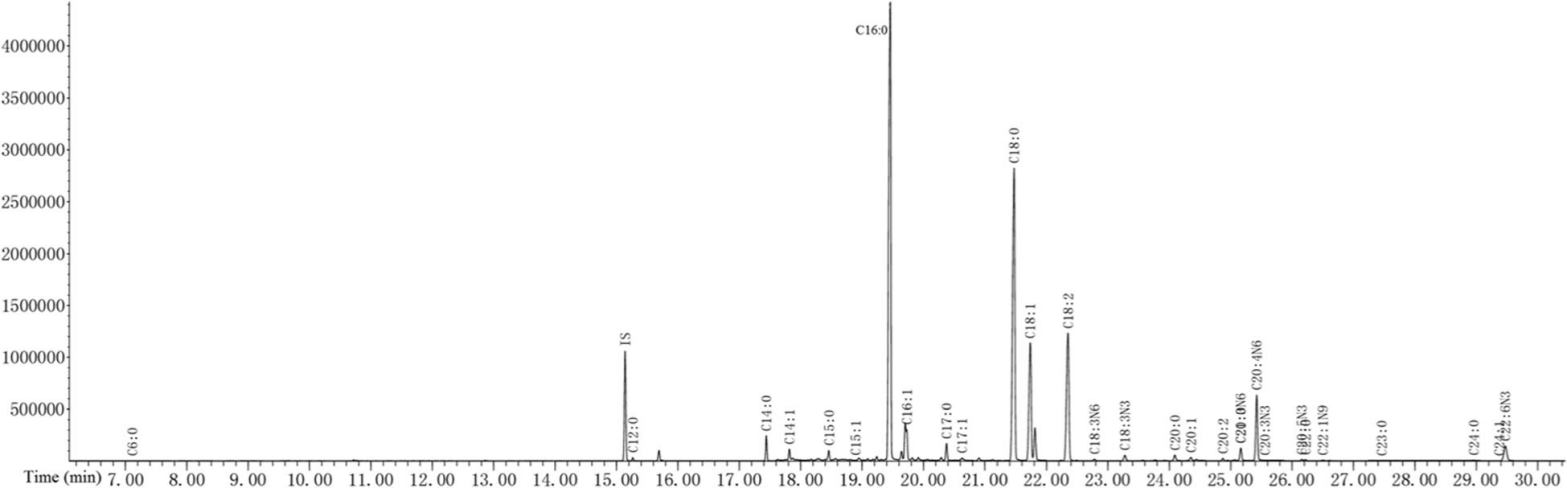
Total ion flow chromatography of liver samples (TIC)

By scanning and analyzing the established fatty acid analysis method, it is found that the internal standard IS separated from the standard products at the time of 1000 mg·L^− 1^ concentration, which indicates that the method is reliable. Figure 1 is the total ion chromatography at the 1000 mg·L^− 1^ concentration, from which it can be seen that the fatty acids are separated, and a total of 34 fatty acids are obtained at this concentration, of which 2 fatty acids are not separated due to their isomers and 1 are not detected. Details are shown in Fig. 1. Using the same method to analyze liver samples, it was found that fatty acids such as C8:0, C10:0, C11:0, C13:0 in standard products were not detected in liver samples, but other fatty acids could be clearly seen on the graph (Fig. 2). Details are shown in Fig. 2.

### Standard curves and linear ranges

The concentration series of standard products were tested by GC-MS respectively, with the concentration of each component of standard product as the horizontal coordinate, the ratio of the concentration of each component of the standard product to the peak area of the inner standard was longitudinal coordinate, and the linearity and correlation coefficient were calculated for each component according. The linear regression equations of methyl ester components of each fatty acid are shown in Table 2. The correlation coefficients are all > 0.99.

**Table 2:** Linear regression equations for standard components of methyl ester of 34 fatty acids

Number	Name abbreviated	Retention time(min)	Quantitative ions(m/z)	Linear equation	Correlation coefficient (R ^2^)	Linear range (mg/L)
1	C6:0	7.075	74	Y = 0.05665x-0.01393	0.9993	0.0263–26.3
2	C8:0	10.166	74	Y = 0.06949x + 0.006365	0.9990	0.0263–26.3
3	C10:0	12.867	74	Y = 0.07117x + 0.01177	0.9980	0.0263–26.3
4	C11:0	14.101	74	Y = 0.07200x + 0.01237	0.9978	0.0263–26.3
5	C12:0	15.272	74	Y = 0.07187x + 0.01333	0.9972	0.0263–26.3
6	C13:0	16.384	74	Y = 0.07259x + 0.009960	0.9979	0.0263–26.3
7	C14:0	17.446	74	Y = 0.07177x + 0.007484	0.9981	0.0263–26.3
8	C14:1	17.844	55	Y = 0.02616x + 0.006221	0.9970	0.0263–26.3
9	C15:0	18.463	74	Y = 0.07181x + 0.002304	0.9988	0.0263–26.3
10	C15:1	18.848	55	Y = 0.02673x + 0.0007506	0.9988	0.0263–26.3
11	C16:0	19.442	74	Y = 0.05832x + 0.03077	0.9944	0.0526–52.6
12	C16:1	19.703	55	Y = 0.02076x + 0.04067	0.9961	0.0263–26.3
13	C17:0	20.379	74	Y = 0.06140x-0.005180	0.9993	0.0263–26.3
14	C17:1	20.667	55	Y = 0.02095x + 0.003006	0.9992	0.0263–26.3
15	C18:0	21.453	74	Y = 0.05421x-0.009999	0.9994	0.0263–26.3
16	C18:1	21.729	55	Y = 0.01613x-0.002003	0.9990	0.0526–52.6
17	C18:2	22.343	67	Y = 0.01982x-0.009295	0.9993	0.0526–52.6
18	C18:3 N6	22.785	79	Y = 0.02045x-0.007422	0.9985	0.0263–26.3
19	C18:3 N3	23.279	79	Y = 0.02420x-0.01029	0.9978	0.0263–26.3
20	C20:0	24.091	74	Y = 0.03425x-0.01337	0.9981	0.0263–26.3
21	C20:1	24.349	55	Y = 0.01309x-0.004675	0.9984	0.0263–26.3
22	C20:2	24.873	81	Y = 0.01390x-0.007475	0.9975	0.1315–26.3
23	C21:0	25.146	74	Y = 0.02540x-0.009271	0.9984	0.0263–26.3
24	C20:3 N6	25.161	67	Y = 0.01257x-0.004708	0.9984	0.0263–26.3
25	C20:4 N6	25.417	79	Y = 0.01483x-0.007204	0.9965	0.0263–26.3
26	C20:3 N3	25.554	79	Y = 0.01606x-0.007747	0.9959	0.0263–26.3
27	C20:5 N3	26.159	79	Y = 0.01543x-0.01027	0.9958	0.1315–26.3
28	C22:0	26.212	74	Y = 0.01714x-0.007112	0.9964	0.0263–26.3
29	C22:1 N9	26.494	55	Y = 0.007372x-0.003041	0.9924	0.0263–26.3
30	C22:2	27.123	67	Y = 0.008102x-0.004111	0.9959	0.263–26.3
31	C23:0	27.450	74	Y = 0.01109x-0.006266	0.9936	0.0263–26.3
32	C24:0	28.928	74	Y = 0.007231x-0.002510	0.9953	0.263–26.3
33	C24:1	29.357	55	Y = 0.003275x-0.002131	0.9919	0.0263–26.3
34	C22:6 N3	29.462	79	Y = 0.002571x-0.00006972	0.9990	0.0263–26.3

### Determination of fatty acid content in liver samples

**Fig. 3:**
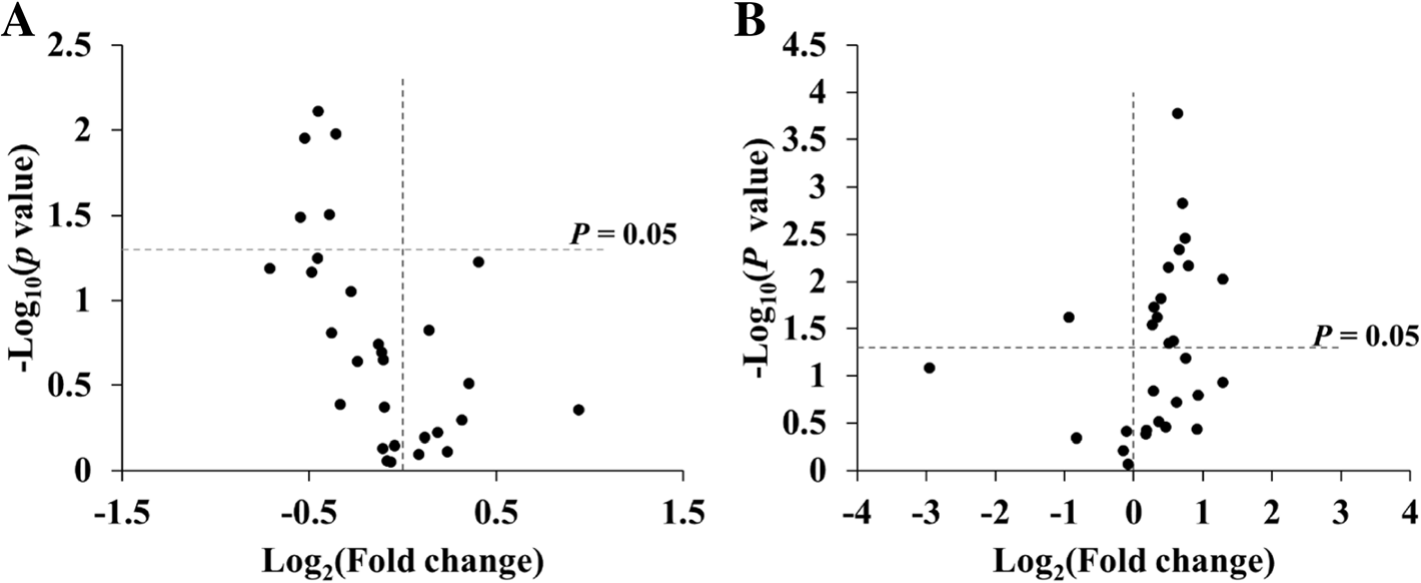
Volcanic map analysis of fatty acid content in liver samples (**a**: Represents a comparison of blank and aging models; **b**: Represents a comparison of aging and Middle Dose group of PMT)

**Fig. 4:**
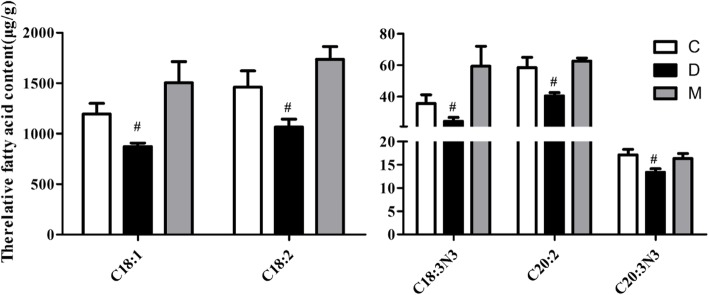
Column Chart of 5 fatty acids in Aged mice. (#: Compared with C group and PMT-M group *P* < 0.05)

The liver samples were tested by GC-MS as the above analysis method, and the overall change trend of all fatty acids was analyzed by volcanic map (Fig. 3). As can be seen from Fig. 3 A, most of the fatty acids decreased during the establishment of the aging model, of which 5 fatty acids were statistically significant (*P* < 0.05); As shown in Fig. 3 B, after the administration of the drug, most of the fatty acids increased, of which 14 fatty acids were statistically significant. Details are shown in Fig. 3. Table 3 lists the fatty acid content of the samples measured, and it is clear from the data that the 5 fatty acids lowered in the process of establishing an aging model in D-galactose are C18:1 (oleic acid), C18:2 (linoleic acid), C18:3 N3 (linolenic acid), C20:2 (20 methyl phthalate), C20: 3 N3 (methyl ester 20 carbon propionate), and these fatty acids begin to rise after the action of PMT (*P* < 0.05); And in the process of establishing the aging model, a number of fatty acids were found to have been lowered (although there was no difference), such as C20:3 N6, C20:4 N6 (arachidonic acid), C23:0, C24:0, C22:6 N3, etc., but after the action of PMT, these fatty acids began to increase and appear different (*P* < 0.05). The effect of PMT on fatty acids was analyzed by column chart, which made it clearer, as shown in Fig. 4. This results show that PMT can improve the changes of fatty acids such as C18:1, C18:2, C18:3 N3, C20:2 and C20:3 N3 caused by aging. Details are shown in Table 3.

**Table 3:** Average fatty acid content of each group in liver samples(μg·g^− 1^)

Name	C	D	M	P1	P2
C6:0	5.630	6.197	5.743	0.149	0.383
C8:0	ND	ND	ND	–	–
C10:0	ND	ND	ND	–	–
C11:0	ND	ND	ND	–	–
C12:0	3.157	3.927	7.390	0.504	0.367
C13:0	ND	ND	ND	–	–
C14:0	33.300	36.097	49.707	0.636	0.347
C14:1	59.640	70.243	39.407	0.771	0.448
C15:0	20.413	19.250	36.547	0.874	0.160
C15:1	5.227	10.033	1.293	0.440	0.081
C16:0	1594.823	1482.047	1873.903	0.223	0.024
C16:1	324.407	429.163	223.873	0.059	0.024
C17:0	66.237	52.497	80.517	0.409	0.190
C17:1	21.893	20.300	49.370	0.739	0.117
C18:0	1244.757	1136.947	1365.090	0.181	0.029
C18:1	1193.853	872.173	1504.917	0.008	0.007
C18:2	1401.503	1066.510	1735.007	0.031	0.001
C18:3 N6	18.937	21.530	27.560	0.596	0.304
C18:3 N3	35.637	24.373	59.337	0.032	0.009
C20:0	25.517	32.553	29.170	0.306	0.612
C20:1	45.880	38.770	55.160	0.228	0.045
C20:2	58.337	40.490	62.637	0.011	0.000
C21:0	26.010	24.293	27.543	0.422	0.373
C20:3 N6	184.230	131.490	195.570	0.068	0.042
C20:4 N6	1443.350	1051.317	1483.187	0.056	0.007
C20:3 N3	17.120	13.367	16.340	0.010	0.018
C20:5 N3	30.183	32.023	53.893	0.797	0.065
C22:0	33.040	32.043	38.870	0.712	0.145
C22:1 N9	22.250	21.280	20.107	0.887	0.850
C23:0	15.323	12.630	16.520	0.088	0.015
C24:0	33.510	25.720	40.503	0.155	0.005
C24:1	16.950	15.657	17.643	0.201	0.411
C22:6 N3	2931.230	1787.987	2988.343	0.064	0.003

Note: C: Control group, D: Model group, M: Middle dose Group of PMT, P1: *P* value compared to control group and model group, P2: *P* value compared to model group and drug delivery group. *ND* not detected

## Discussion

The subacute senescence model established by D-galactose is the main animal model to mimic aging in human being, which has the advantages of low cost, short modeling period and stable result. There are many viewpoints on the causes of senescence induced by D-galactose, among which the main idea is that D-galactose acts from galactose oxidase in the body to produce aldohexose and hydrogen peroxide, which increases reactive oxygen species and lipid peroxidation, and eventually produces oxygen free radicals, which leads to aging of the body [12]. Oxygen free radicals are harmful substances produced by the body, which can compete for other substances of electrons (such as proteins), so that its material structure changes, and the absence of electronic substances to deprive adjacent electrons, and ultimately lead to the corresponding disease [13, 14].

The essence of aging in organisms is the excess generation of free radicals and abnormal chain reaction. Excessive free radicals in the body can attack the unsaturated fatty acids on the biofilm of the body, so that the body produces lipid peroxidation, the formation of damage to the body’s lipid peroxides, such as aldehydes (malondialdehyde), hydrogen oxygen base, etc. [15]. MDA, one of the main products of lipid peroxidation, is a sensitive index to measure the free radicals of the body, due to it can objectively reflect the level of free radicals of the body, the degree of lipid peroxidation and damage [16, 17]. GSH-Px is an important enzyme in the body that catalyzes the decomposition of hydrogen peroxide, and it is also an important antioxidant and free radical scavenger in the body. GSH-Px specifically catalyze the reduction response of glutathione to hydrogen peroxide, which can protect the integrity of cell membranes and functions [18, 19]. SOD, an important antioxidant enzyme in the body, is a natural free radical scavenging factor in the body, which can transform harmful free radicals into hydrogen peroxide, and its vitality indirectly reflects the body’s free radical scavenging ability. Therefore, combined with the test results of enzyme activity including MDA, GSH-Px and SOD, the aging process of the body can be analyzed effectively. In this study, the MDA content of liver increased obviously and the activity of SOD and GSH-Px decreased obviously in aging model mice established by D-galactose. However, after the administration of PMT, the antioxidant enzyme activity of the liver was obviously restored, and the content of MDA was obviously reduced. These results showed that PMT delayed the aging, at least partly, through enhancing the antioxidant enzyme activity.

Indeed, the PMT itself has a certain toxicity, but the use of small doses is more beneficial than its disadvantages. Some studies have pointed out that PMT may damage liver tissue, mainly due to the long-term or higher doses use of PMT, which may lead to the body cannot timely removal of toxic and harmful substances, and then affected by the body tissue [20]. Reactive oxygen species (ROS) is a harmful substance present in the body and can be removed under the action of antioxidant enzymes such as SOD, GSH-Px. When toxic and harmful substances accumulate exceeding the body’s removal threshold, resulting in a decrease in enzyme activity such as SOD, GSH-Px. The results showed that the main component (styrene glycosides) of PMT improved the neuronal damage induced by oxygen–glucose deprivation followed by reperfusion (OGD-R) and reduce the production of ROS in cells [11]. Therefore, Polygonum can remove ROS and other harmful substances in the body, so as to achieve the protective effect on the body.

As the aging process occurs, many of the body’s functions and ingredients will change, including fatty acids. Previous study indicates that there is a relationship between fatty acids and cognitive function and dementia, and that proper intake of essential fatty acids has the effect of preventing cognitive decline [21]. Moreover, when comparing and analyzing the serum metabolites of young mice and senile mice by liquid chromatography series mass spectrometry, it was found that several unsaturated fatty acids decreased in senescent mice, including linoleic acid, linolenic acid, arachidonic acid and so on [22]. Using GC/TOFMS technique to analyze the liver of 12 weeks and 20 weeks rats, it was also found that fatty acids such as arachidonic acid decreased with the increase of age [23]. The decrease of these unsaturated fatty acids may be related to the increase of free radicals in aging and lipid peroxidation in cell membranes [24]. Arachidonic acid can be metabolized into a variety of bioactive substances in vivo, such as thrombotic TXA2, prostaglandin PGl2, leukotriene LTB4 and so on. These substances have anti-inflammatory, hypolipidemia, immunomodulatory and other effects, and in its chemical structure of the unsaturated bond can capture free radicals, and eliminate the harmful effects of free radicals. In this study, the metabolism of fatty acids in the liver was discussed using the role of PMT to delay senescence, and it was found that PMT could regulate the changes of multiple fatty acids, including the polyunsaturated fatty acids needed by the body: C18:2 (linoleic acid), C18:3 N3 (linolenic acid), C20:4 N6 (arachidonic acid) and so on. And in the relevant literature, it is pointed out that PMT has anti-inflammatory, lowering blood lipids, enhance immunity and other effects [8, 9, 25], as well as the removal of free radicals, reduce lipid peroxidation and other effects [26, 27]. Therefore, it is speculated that PMT can regulate the metabolism of unsaturated fatty acids such as arachidonic acid.

Linolenic acid has multiple effects, including anti-inflammatory, antioxidant and nerve protection, et al. [28]. Linoleic acid is essential to ensure the normal metabolism of cholesterol, such as the lack of linoleic acid, it makes cholesterol metabolic disorders, easy to deposit in the wall of blood vessels, and then cause vascular diseases, so linoleic acid has the role of preventing atherosclerosis. In the relevant studies, it was found that proper supplementation of fatty acids such as linoleic acid and linolenic acid can indeed improve cognitive impairment or memory loss caused by aging [29, 30]. As a metabolic product of arachidonic acid, C20:3 N3 are characterized by anti-inflammatory and fibrinolytic effects [31, 32].

## Conclusion

PMT exerted beneficial effects on D-galactose-induced subacute aging model in mice via significantly improving the content of fatty acids. It’s mechanism, at least partly, through mediating the metabolism of fatty acids including C18:1, C18:2, C18:3 N3, C20:2 and C20:3 N3. However, the precise molecular mechanism of PMT needs to explore in further study.

## References

[1] Vitetta L, Linnane AW (2014). Endocellular regulation by free radicals and hydrogen peroxide: key determinants of the inflammatory response[J]. Inflammopharmacology..

[2] Fusco D, Colloca G, Lo Monaco MR (2007). Effects of antioxidant supplementation on the aging process[J]. Clin Interv Aging.

[3] Keller JN (2006). Age-related neuropathology, cognitive decline, and Alzheimer's disease[J]. Ageing Res Rev.

[4] Gong Z, Tas E, Yakar S (2017). Hepatic lipid metabolism and non-alcoholic fatty liver disease in aging[J]. Mol Cell Endocrinol.

[5] Verdier V, Csardi G, de Preux-Charles AS (2012). Aging of myelinating glial cells predominantly affects lipid metabolism and immune response pathways[J]. Glia..

[6] Yan S, Wu B, Lin Z (2009). Metabonomic characterization of aging and investigation on the anti-aging effects of total flavones of Epimedium[J]. Mol BioSyst.

[7] Montoliu I, Scherer M, Beguelin F (2014). Serum profiling of healthy aging identifies phospho- and sphingolipid species as markers of human longevity[J]. Aging.

[8] Zhang MS, Liu X, Meng XM (2012). Antioxidative and antimicrobial activities of Polygonum multiflorum Thunb. in vitro[J]. Food Sci Technol.

[9] Um MY, Choi WH, Aan JY (2006). Protective effect of Polygonum multiflorum Thunb on amyloid beta-peptide 25-35 induced cognitive deficits in mice[J]. J Ethnopharmacol.

[10] Zhou X, Yang Q, Xie Y (2015). Tetrahydroxystilbene glucoside extends mouse life span via upregulating neural klotho and downregulating neural insulin or insulin-like growth factor 1[J]. Neurobiol Aging.

[11] Wang T, Gu J, Wu PF (2009). Protection by tetrahydroxystilbene glucoside against cerebral ischemia: involvement of JNK, SIRT1, and NF-kappaB pathways and inhibition of intracellular ROS/RNS generation[J]. Free Radic Biol Med.

[12] Ho SC, Liu JH, Wu RY (2003). Establishment of the mimetic aging effect in mice caused by D-galactose[J]. Biogerontology.

[13] Dizdaroglu M, Jaruga P (2012). Mechanisms of free radical-induced damage to DNA[J]. Free Radic Res.

[14] Liochev SI (2013). Reactive oxygen species and the free radical theory of aging[J]. Free Radic Biol Med.

[15] Halliwell B (2009). The wanderings of a free radical[J]. Free Radic Biol Med.

[16] Shen Y, Zhang H, Cheng L (2016). In vitro and in vivo antioxidant activity of polyphenols extracted from black highland barley[J]. Food Chem.

[17] Barrera G, Pizzimenti S, Daga M (2018). Lipid peroxidation-derived aldehydes, 4-Hydroxynonenal and malondialdehyde in aging-related disorders[J]. Antioxidants (Basel).

[18] Edith AR, Adolfo RG (2016). Reactive oxygen species production and antioxidant enzyme activity during epididymal sperm maturation in Corynorhinus mexicanus bats[J]. Reprod Biol.

[19] Lei L, Ou L, Yu X (2016). The antioxidant effect of Asparagus cochinchinensis (Lour.) Merr. Shoot in d-galactose induced mice aging model and in vitro[J]. J Chin Med Assoc.

[20] Lei X, Chen J, Ren J (2015). Liver Damage Associated with *Polygonum multiflorum* Thunb.: A Systematic Review of Case Reports and Case Series [J]. Evid Based Complement Alternat Med.

[21] Cederholm T, Jr SN, Palmblad J (2013). ω-3 fatty acids in the prevention of cognitive decline in humans[J]. Adv Nutr.

[22] Hao XW, Lu HT, Xia DM (2017). Serum metabolites profile analysis of wild-type C57BL/6 senile mice based on liquid chromatography tandem mass spectrometry[J]. Chin J Gerontol.

[23] Huang Q, Lu YH, Wang GJ (2009). Metabonomic profiling of plasma metabolites in Wistar rats to study the effect of aging by means of GC/TOFMS-based techniques[J]. Yao Xue Xue Bao.

[24] Liu HM, Luo ZS, Li XF (2002). The relationship between metabolic changes of unsaturated fatty acids and senescence in rats [J]. Chin J Gerontol.

[25] Lv L, Cheng Y, Zheng T (2014). Purification, antioxidant activity and antiglycation of polysaccharides from Polygonum multiflorum Thunb[J]. Carbohydr Polym.

[26] Ip SP, Tse ASM, Poon MKT (2015). Antioxidant activities of Polygonum multiflorum Thunb. In vivo and in vitro[J]. Phytother Res.

[27] Zhou Y, Liu G, Feng J (2014). Study on separation and DPPH radical scavenging activities of 3 components from Polygonum multiflorum Thunb[J]. J Guangdong Pharmaceutical Univ.

[28] Kim KB, Nam YA, Kim HS (2014). α-linolenic acid: nutraceutical, pharmacological and toxicological evaluation[J]. Food Chem Toxicol.

[29] Jenkins NDM, Housh TJ, Miramonti AA (2016). Effects of rumenic acid rich conjugated linoleic acid supplementation on cognitive function and handgrip performance in older men and women[J]. Exp Gerontol.

[30] Gao H, Yan P, Zhang S (2016). Chronic alpha-linolenic acid treatment alleviates age-associated neuropathology: roles of PERK/eIF2α signaling pathway[J]. Brain Behav Immun.

[31] Spector AA, Fang X, Snyder GD (2004). Epoxyeicosatrienoic acids (EETs): metabolism and biochemical function[J]. Prog Lipid Res.

[32] Oltman CL, Weintraub NL, Vanrollins M (1998). Epoxyeicosatrienoic acids and dihydroxyeicosatrienoic acids are potent vasodilators in the canine coronary microcirculation[J]. Circ Res.

